# Supplementary material for: The Detection of CMV in Saliva Can Mark a Systemic Infection with CMV in Renal Transplant Recipients
Source: Int J Mol Sci. 2019 Oct 22;20(20):5230. doi: 10.3390/ijms20205230 (PMC6829882; doi:10.3390/ijms20205230)
Supplement: Supplementary file 1 [file ijms-20-05230-s001.pdf]

**Supplementary Table S1. Comparison of demographics, immunological measures of CMV, biomarkers and vascular health in RTR with and without detectable CMV DNA.**

| | *A* | *B* | *A v B* | *C* | *D* | *C v D* |
|---|---|---|---|---|---|---|
| | **Saliva CMV DNA Positive RTR (n = 11)** | **Saliva CMV DNA Negative RTR (n = 71)** | ***p*-Value[a]** | **Plasma CMV DNA Positive RTR (n = 16)** | **Plasma CMV DNA Negative RTR (n = 61)** | ***p*-Value[a]** |
| **Demographics** | | | | | | |
| **Age (years)** | 58 (31–76) | 57 (31–73) | 0.50 | 59 (31–76) | 64 (31–73) | 0.67 |
| **Male/ Female** | 7/4 | 39/32 | 0.75 [b] | 12/4 | 32/29 | 0.16[b] |
| **Immunological measures of CMV** | | | | | | |
| **CMV Seropositive** | 10 (91%) | 59 (83%) | | 16 (100%) | 53 (87%) | |
| **CMV Seronegative** | 1 (9%) | 12 (17%) | | 0 (0%) | 8 (13%) | |
| **CMV lysate antibodies (AU)** | 797 (1–5582) | 554 (0–7611) | 0.26 | 810 (76–5582) | 597 (0–7611) | 0.19 |
| **gB antibodies (AU)** | 527 (0–2932) | 231 (0–2035) | **0.009** | 414 (144–2932) | 238 (0–2035) | **0.006** |
| **IE-1 antibodies (AU)** | 90 (17–1446), | 98 (5–4775) | 0.10 | 128 (5–4775) | 99 (12–3646) | 0.44 |
| **CMV lysate T-cells[c]** | 63 (0–938), n = 9 | 60 (0–2077), n = 56 | 0.99 | 63 (0–938), n = 13 | 139 (0–2077), n = 47 | 0.86 |
| **IE-1 pooled peptides T-cells[c]** | 210 (1–1533), n = 9 | 50 (0-1888), n = 55 | 0.14 | 210 (10–1533), n = 13 | 54.5 (0–1888), n = 46 | **0.04** |
| **VLE peptide T-cells[c]** | 184 (0–1160), n = 6 | 1 (0–693), n = 27 | **0.02** | 184 (0–1160), n = 6 | 2 (0–693), n = 27 | 0.16 |
| **pp65 pooled peptides T-cells[c]** | 283 (0–1989), n = 9 | 358 (0–1963), n = 56 | 0.93 | 666 (0–1989), n = 13 | 361 (0–1963), n = 47 | 0.22 |
| **NLV peptide T-cells[c]** | 821 (10–1336), n = 5 | 22 (0–1896), n = 27 | **0.03** | 487 (0–985), n = 5 | 50 (0–1896), n = 27 | 0.54 |
| **V$\delta$2$^-$ $\gamma\delta$ T-cells[d]** | 4.23 (0.19–14.30), n = 11 | 1.17 (0.06–17.50), n = 69 | **0.013** | 4.23 (0.26–14.30), n = 15 | 1.19 (0.07–17.50), n = 60 | **0.005** |
| **Inflammatory** Biomarkers | | | | | | |
| **sTNFR1, pg/mL** | 9065 (6062–14385) | 3431 (410–33000) | 0.63 | 9219 (4349–20447) | 3411 (410–33000) | 0.35 |
| **sCD14, ng/mL** | 2137 (1623–2546) | 2032 (1384–3640) | 0.97 | 2063 (1487–813) | 2040 (1384–3640) | 0.56 |
| **CRP, µg/mL** | 1.89 (0.05–13.38) | 1.47 (0.12–20.22) | 0.55 | 2.45 (0.05–13.38) | 1.47 (0.12–20.22) | 0.36 |
| **Vascular** Biomarkers | | | | | | |
| **VCAM-1, ng/mL** | 498 (317–719) | 439 (249–1143) | 0.27 | 533 (317–727) | 431 (264–1143) | **0.03** |
| **ICAM-1, ng/mL** | 153 (86–182) | 133 (89–330) | 0.20 | 164 (86–205) | 133 (89–330) | 0.08 |
| **P-selectin, ng/mL** | 39 (27–78) | 53 (28–107) | **0.010** | 39 (27–83) | 52 (28–107) | **0.010** |
| **Assessments of** Vascular Health | | | | | | |
| **FMD** | 3.2 (0.1–5.1), n = 10 | 4.2 (0–15.8), n = 61 | 0.087 | 3.2 (0.1–5.1), n = 15 | 4.4 (0–15.8), n = 52 | 0.062 |
| **Left cIMT (mm)** | 0.63 (0.45–0.95), n = 10 | 0.66 (0.44–1.30), n = 63 | 0.80 | 0.63 (0.45–0.95), n = 15 | 0.82 (0.44–1.30), n = 54 | 0.75 |
| **Right cIMT (mm)** | 0.61 (0.43–0.91), n = 10 | 0.65 (0.45–1.33), n = 63 | 0.67 | 0.61 (0.43–1.0), n = 15 | 0.75 (0.45–1.33), n = 54 | 0.52 |

[a] Mann- Whitney test shown as median (range), [b] Fisher's exact test, [c] expressed as IFN$\gamma$ spot forming units per 200,000 cells, [d] expressed as a % of CD3 T-cells
